# Supplementary material for: Tumor-Infiltrating Lymphocytes (TILs) in Epithelial Ovarian Cancer: Heterogeneity, Prognostic Impact, and Relationship with Immune Checkpoints
Source: Cancers (Basel). 2022 Oct 29;14(21):5332. doi: 10.3390/cancers14215332 (PMC9656626; doi:10.3390/cancers14215332)
Supplement: Supplementary file 1 [file cancers-14-05332-s001.zip › cancers-1929643-supplementary.pdf]

Supplementary Materials File S1. Search strategy

MEDLINE with OVID:

exp Tumor Microenvironment/ or exp CD8-Positive T-Lymphocytes/ or exp Lymphocytes,Tumor-Infiltrating/ or exp T-Lymphocytes/ or ("tumor* microenvironment*" or "T Lymphocyte*" or "CD8-Positive T-lymphocyte*").ab,kf,ti. or exp CD4-Positive T-Lymphocytes/ or exp B-Lymphocytes/ or exp T-Lymphocytes, Helper-Inducer/ or exp T-Lymphocytes, Regulatory/ or ("CD4-Positive T-Lymphocyte*" or "immunocheckpoint*" or "B-Lymphocyte*" or "follicular helper" or "follicular helper T" or "lymphocyte* regulator* or "regulator* T" or "tfh" or "th1" or "th2" or "th17" or "treg" or "tils").ab,kf,ti.

AND

exp Ovarian Neoplasms/ or exp Carcinoma, Ovarian Epithelial/ or ("ovarian* neoplasm*" or "ovarian* cancer*" or "epithelial* ovarian* cancer* " or "high grade serous ovarian cancer*" or "low grade serous ovarian cancer*" or "endometrioid ovarian cancer*" or "clear* cell* ovarian cancers*" or "mucinous ovarian cancer*" or "ovarian carcinosarcoma").ab,kf,ti.

EMBASE:

'tumor microenvironment'/exp/mj OR 'CD8+ T lymphocyte'/exp/mj OR 'tumor associated leukocyte'/exp/mj OR 'CD4+ T lymphocyte'/exp/mj OR 'helper cell'/exp/mj OR 'Th1 cell'/exp/mj OR 'Th2 cell'/exp/mj OR 'Th17 cell'/exp/mj OR 'regulatory T lymphocyte'/exp/mj OR 'B lymphocyte'/exp/mj OR ('cd4-positive t-lymphocyte*' OR 'immunocheckpoint*' OR 'b-lymphocyte*' OR 'follicular helper' OR 'follicular helper t' OR 'tumor* microenvironment*' OR 't lymphocyte*' OR 'lymphocyte* regulator*' OR 'regulator* t' OR 'cd8-positive t-lymphocyte*' OR 'tfh' OR 'th1' OR 'th2' OR 'th17' OR 'treg' OR 'tils'):ti,ab,kw

AND

'ovary carcinoma'/exp/mj OR 'high grade serous ovarian cancer'/exp OR 'ovary tumor'/exp/mj OR 'low grade serous ovarian cancer'/exp OR 'endometrioid ovarian cancer'/exp OR 'clear cell ovarian cancer'/exp OR 'mucinous ovarian cancer'/exp OR 'ovarian carcinosarcoma'/exp OR ('ovarian* neoplasm*' OR 'ovarian* cancer*' OR 'epithelial* ovarian* cancer*' OR 'high grade serous ovarian cancer*' OR 'low grade serous ovarian cancer*' OR 'endometrioid ovarian cancer*' OR 'clear* cell* ovarian cancers*' OR 'mucinous ovarian cancer*' OR 'ovarian carcinosarcoma'):ti,ab,kw
